# Predicting 90-day survival of patients with COVID-19: Survival of Severely Ill COVID (SOSIC) scores

**DOI:** 10.1186/s13613-021-00956-9

**Published:** 2021-12-11

**Authors:** Matthieu Schmidt, Bertrand Guidet, Alexandre Demoule, Maharajah Ponnaiah, Muriel Fartoukh, Louis Puybasset, Alain Combes, David Hajage, for the COVID-ICU Investigators, Alain Mercat, Pierre Asfar, François Beloncle, Julien Demiselle, Tài Pham, Arthur Pavot, Xavier Monnet, Christian Richard, Alexandre Demoule, Martin Dres, Julien Mayaux, Alexandra Beurton, Cédric Daubin, Richard Descamps, Aurélie Joret, Damien Du Cheyron, Frédéric Pene, Jean-Daniel Chiche, Mathieu Jozwiak, Paul Jaubert, Guillaume Voiriot, Muriel Fartoukh, Marion Teulier, Clarisse Blayau, Erwen L’Her, Cécile Aubron, Laetitia Bodenes, Nicolas Ferriere, Johann Auchabie, Anthony Le Meur, Sylvain Pignal, Thierry Mazzoni, Jean-Pierre Quenot, Pascal Andreu, Jean-Baptiste Roudau, Marie Labruyère, Saad Nseir, Sébastien Preau, Julien Poissy, Daniel Mathieu, Sarah Benhamida, Rémi Paulet, Nicolas Roucaud, Martial Thyrault, Florence Daviet, Sami Hraiech, Gabriel Parzy, Aude Sylvestre, Sébastien Jochmans, Anne-Laure Bouilland, Mehran Monchi, Marc Danguy des Déserts, Quentin Mathais, Gwendoline Rager, Pierre Pasquier, Reignier Jean, Seguin Amélie, Garret Charlotte, Canet Emmanuel, Jean Dellamonica, Clément Saccheri, Romain Lombardi, Yanis Kouchit, Sophie Jacquier, Armelle Mathonnet, Mai-Ahn Nay, Isabelle Runge, Frédéric Martino, Laure Flurin, Amélie Rolle, Michel Carles, Rémi Coudroy, Arnaud W. Thille, Jean-Pierre Frat, Maeva Rodriguez, Pascal Beuret, Audrey Tientcheu, Arthur Vincent, Florian Michelin, Fabienne Tamion, Dorothée Carpentier, Déborah Boyer, Christophe Girault, Valérie Gissot, Stéphan Ehrmann, Charlotte Salmon Gandonniere, Djlali Elaroussi, Agathe Delbove, Yannick Fedun, Julien Huntzinger, Eddy Lebas, Grâce Kisoka, Céline Grégoire, Stella Marchetta, Bernard Lambermont, Laurent Argaud, Thomas Baudry, Pierre-Jean Bertrand, Auguste Dargent, Christophe Guitton, Nicolas Chudeau, Mickaël Landais, Cédric Darreau, Alexis Ferre, Antoine Gros, Guillaume Lacave, Fabrice Bruneel, Mathilde Neuville, Jérôme Devaquet, Guillaume Tachon, Richard Gallot, Riad Chelha, Arnaud Galbois, Anne Jallot, Ludivine Chalumeau Lemoine, Khaldoun Kuteifan, Valentin Pointurier, Louise-Marie Jandeaux, Joy Mootien, Charles Damoisel, Benjamin Sztrymf, Matthieu Schmidt, Alain Combes, Juliette Chommeloux, Charles-Edouard Luyt, Frédérique Schortgen, Leon Rusel, Camille Jung, Florent Gobert, Damien Vimpere, Lionel Lamhaut, Bertrand Sauneuf, Liliane Charrrier, Julien Calus, Isabelle Desmeules, Benoît Painvin, Jean-Marc Tadie, Vincent Castelain, Baptiste Michard, Jean-Etienne Herbrecht, Mathieu Baldacini, Nicolas Weiss, Sophie Demeret, Clémence Marois, Benjamin Rohaut, Pierre-Henri Moury, Anne-Charlotte Savida, Emmanuel Couadau, Mathieu Série, Nica Alexandru, Cédric Bruel, Candice Fontaine, Sonia Garrigou, Juliette Courtiade Mahler, Maxime Leclerc, Michel Ramakers, Pierre Garçon, Nicole Massou, Ly Van Vong, Juliane Sen, Nolwenn Lucas, Franck Chemouni, Annabelle Stoclin, Alexandre Avenel, Henri Faure, Angélie Gentilhomme, Sylvie Ricome, Paul Abraham, Céline Monard, Julien Textoris, Thomas Rimmele, Florent Montini, Gabriel Lejour, Thierry Lazard, Isabelle Etienney, Younes Kerroumi, Claire Dupuis, Marine Bereiziat, Elisabeth Coupez, François Thouy, Clément Hoffmann, Nicolas Donat, Anne Chrisment, Rose-Marie Blot, Antoine Kimmoun, Audrey Jacquot, Matthieu Mattei, Bruno Levy, Ramin Ravan, Loïc Dopeux, Jean-Mathias Liteaudon, Delphine Roux, Brice Rey, Radu Anghel, Deborah Schenesse, Vincent Gevrey, Jermy Castanera, Philippe Petua, Benjamin Madeux, Otto Hartman, Michael Piagnerelli, Anne Joosten, Cinderella Noel, Patrick Biston, Thibaut Noel, Gurvan Bouar, Messabi Boukhanza, Elsa Demarest, Marie-France Bajolet, Nathanaël Charrier, Audrey Quenet, Cécile Zylberfajn, Nicolas Dufour, Buno Mégarbane, Sqébastian Voicu, Nicolas Deye, Isabelle Malissin, François Legay, Matthieu Debarre, Nicolas Barbarot, Pierre Fillatre, Bertrand Delord, Thomas Laterrade, Tahar Saghi, Wilfried Pujol, Pierre-Julien Cungi, Pierre Esnault, Mickael Cardinale, Vivien Hong Tuan Ha, Grégory Fleury, Marie-Ange Brou, Daniel Zafimahazo, David Tran-Van, Patrick Avargues, Lisa Carenco, Nicolas Robin, Alexandre Ouali, Lucie Houdou, Christophe Le Terrier, Noémie Suh, Steve Primmaz, Jérome Pugin, Emmanuel Weiss, Tobias Gauss, Jean-Denis Moyer, Catherine Paugam Burtz, Béatrice La Combe, Rolland Smonig, Jade Violleau, Pauline Cailliez, Jonathan Chelly, Antoine Marchalot, Cécile Saladin, Christelle Bigot, Pierre-Marie Fayolle, Jules Fatséas, Amr Ibrahim, Dabor Resiere, Rabih Hage, Clémentine Cholet, Marie Cantier, Pierre Trouiler, Philippe Montravers, Brice Lortat-Jacob, Sebastien Tanaka, Alexy Tran Dinh, Jacques Duranteau, Anatole Harrois, Guillaume Dubreuil, Marie Werner, Anne Godier, Sophie Hamada, Diane Zlotnik, Hélène Nougue, Armand Mekontso-Dessap, Guillaume Carteaux, Keyvan Razazi, Nicolas De Prost, Nicolas Mongardon, Olivier Langeron, Eric Levesque, Arié Attias, Charles de Roquetaillade, Benjamin G. Chousterman, Alexandre Mebazaa, Etienne Gayat, Marc Garnier, Emmanuel Pardo, Lea Satre-Buisson, Christophe Gutton, Elise Yvin, Clémence Marcault, Elie Azoulay, Michael Darmon, Hafid Ait Oufella, Geoffroy Hariri, Tomas Urbina, Sandie Mazerand, Nicholas Heming, Francesca Santi, Pierre Moine, Djillali Annane, Adrien Bouglé, Edris Omar, Aymeric Lancelot, Emmanuelle Begot, Gaétan Plantefeve, Damien Contou, Hervé Mentec, Olivier Pajot, Stanislas Faguer, Olivier Cointault, Laurence Lavayssiere, Marie-Béatrice Nogier, Matthieu Jamme, Claire Pichereau, Jan Hayon, Hervé Outin, François Dépret, Maxime Coutrot, Maité Chaussard, Lucie Guillemet, Pierre Goffin, Romain Thouny, Julien Guntz, Laurent Jadot, Romain Persichini, Vanessa Jean-Michel, Hugues Georges, Thomas Caulier, Gaël Pradel, Marie-Hélène Hausermann, Thi My Hue Nguyen-Valat, Michel Boudinaud, Emmanuel Vivier, Sylvène Rosseli, Gaël Bourdin, Christian Pommier, Marc Vinclair, Simon Poignant, Sandrine Mons, Wulfran Bougouin, Franklin Bruna, Quentin Maestraggi, Christian Roth, Laurent Bitker, François Dhelft, Justine Bonnet-Chateau, Mathilde Filippelli, Tristan Morichau-Beauchant, Stéphane Thierry, Charlotte Le Roy, Mélanie Saint Jouan, Bruno Goncalves, Aurélien Mazeraud, Matthieu Daniel, Tarek Sharshar, Cyril Cadoz, Rostane Gaci, Sébastien Gette, Guillaune Louis, Sophe-Caroline Sacleux, Marie-Amélie Ordan, Aurélie Cravoisy, Marie Conrad, Guilhem Courte, Sébastien Gibot, Younès Benzidi, Claudia Casella, Laurent Serpin, Jean-Lou Setti, Marie-Catherine Besse, Anna Bourreau, Jérôme Pillot, Caroline Rivera, Camille Vinclair, Marie-Aline Robaux, Chloé Achino, Marie-Charlotte Delignette, Tessa Mazard, Frédéric Aubrun, Bruno Bouchet, Aurélien Frérou, Laura Muller, Charlotte Quentin, Samuel Degoul, Xavier Stihle, Claude Sumian, Nicoletta Bergero, Bernard Lanaspre, Hervé Quintard, Eve Marie Maiziere, Pierre-Yves Egreteau, Guillaume Leloup, Florin Berteau, Marjolaine Cottrel, Marie Bouteloup, Matthieu Jeannot, Quentin Blanc, Julien Saison, Isabelle Geneau, Romaric Grenot, Abdel Ouchike, Pascal Hazera, Anne-Lyse Masse, Suela Demiri, Corinne Vezinet, Elodie Baron, Deborah Benchetrit, Antoine Monsel, Grégoire Trebbia, Emmanuelle Schaack, Raphaël Lepecq, Mathieu Bobet, Christophe Vinsonneau, Thibault Dekeyser, Quentin Delforge, Imen Rahmani, Bérengère Vivet, Jonathan Paillot, Lucie Hierle, Claire Chaignat, Sarah Valette, Benoït Her, Jennifier Brunet, Mathieu Page, Fabienne Boiste, Anthony Collin, Florent Bavozet, Aude Garin, Mohamed Dlala, Kais Mhamdi, Bassem Beilouny, Alexandra Lavalard, Severine Perez, Benoit Veber, Pierre-Gildas Guitard, Philippe Gouin, Anna Lamacz, Fabienne Plouvier, Bertrand P. Delaborde, Aïssa Kherchache, Amina Chaalal, Jean-Damien Ricard, Marc Amouretti, Santiago Freita-Ramos, Damien Roux, Jean-Michel Constantin, Mona Assefi, Marine Lecore, Agathe Selves, Florian Prevost, Christian Lamer, Ruiying Shi, Lyes Knani, Sébastien Pili Floury, Lucie Vettoretti, Michael Levy, Lucile Marsac, Stéphane Dauger, Sophie Guilmin-Crépon, Hadrien Winiszewski, Gael Piton, Thibaud Soumagne, Gilles Capellier, Jean-Baptiste Putegnat, Frédérique Bayle, Maya Perrou, Ghyslaine Thao, Guillaume Géri, Cyril Charron, Xavier Repessé, Antoine Vieillard-Baron, Mathieu Guilbart, Pierre-Alexandre Roger, Sébastien Hinard, Pierre-Yves Macq, Kevin Chaulier, Sylvie Goutte, Patrick Chillet, Anaïs Pitta, Barbara Darjent, Amandine Bruneau, Sigismond Lasocki, Maxime Leger, Soizic Gergaud, Pierre Lemarie, Nicolas Terzi, Carole Schwebel, Anaïs Dartevel, Louis-Marie Galerneau, Jean-Luc Diehl, Caroline Hauw-Berlemont, Nicolas Péron, Emmanuel Guérot, Abolfazl Mohebbi Amoli, Michel Benhamou, Jean-Pierre Deyme, Olivier Andremont, Diane Lena, Julien Cady, Arnaud Causeret, Arnaud De La Chapelle, Christophe Cracco, Stéphane Rouleau, David Schnell, Camille Foucault, Cécile Lory, Thibault Chapelle, Vincent Bruckert, Julie Garcia, Abdlazize Sahraoui, Nathalie Abbosh, Caroline Bornstain, Pierre Pernet, Florent Poirson, Ahmed Pasem, Philippe Karoubi, Virginie Poupinel, Caroline Gauthier, François Bouniol, Philippe Feuchere, Florent Bavozet, Anne Heron, Serge Carreira, Malo Emery, Anne Sophie Le Floch, Luana Giovannangeli, Nicolas Herzog, Christophe Giacardi, Thibaut Baudic, Chloé Thill, Said Lebbah, Jessica Palmyre, Florence Tubach, David Hajage, Nicolas Bonnet, Nathan Ebstein, Stéphane Gaudry, Yves Cohen, Julie Noublanche, Olivier Lesieur, Arnaud Sément, Isabel Roca-Cerezo, Michel Pascal, Nesrine Sma, Gwenhaël Colin, Jean-Claude Lacherade, Gauthier Bionz, Natacha Maquigneau, Pierre Bouzat, Michel Durand, Marie-Christine Hérault, Jean-Francois Payen

**Affiliations:** 1 Sorbonne Université, Institut National de la Santé et de la Recherche Médicale (INSERM) Unité Mixte de Recherche (UMRS) 1166, Institute of Cardiometabolism and Nutrition, Paris, France; 2 Service de Médecine Intensive–Réanimation, Institut de Cardiologie, iCAN, Institute of Cardiometabolism and Nutrition, Hôpital de la Pitié–Salpêtrière, Assistance Publique–Hôpitaux de Paris (APHP), 47, bd de l’Hôpital, 75651 Paris Cedex 13, France; 3 Sorbonne Université, GRC 30, RESPIRE, APHP, Hôpital Pitié–Salpêtrière, Paris, France; 4 Institut Pierre-Louis d’Epidémiologie et de Santé Publique, APHP, Hôpital Saint-Antoine, INSERM, Service de Réanimation, Sorbonne Université, Paris, France; 5 Service de Pneumologie, Médecine Intensive–Réanimation (Département R3S), Groupe Hospitalier Universitaire APHP-Sorbonne Université, site Pitié–Salpêtrière, Paris, France; 6 Sorbonne Université, INSERM UMRS_1158, Neurophysiologie Respiratoire Expérimentale et Clinique, Paris, France; 7 Service de Médecine Intensive–Réanimation, Hôpital Tenon, Département Médico-Universitaire APPROCHES, APHP, Paris, France; 8 Groupe de Recherche Clinique CARMAS, Université Paris-Est Créteil, Créteil, France; 9 CNRS, INSERM, Laboratoire d’Imagerie Biomédicale, Sorbonne Université, Paris, France; 10 Department of Anesthesiology & Critical Care, APHP, Hôpital Pitié–Salpêtrière, Sorbonne Université, Paris, France; 11 Département de Santé Publique, Centre de Pharmacoépidémiologie (Cephepi), INSER, Institut Pierre-Louis d’Epidémiologie et de Santé Publique, APHP, Hôpital Pitié–Salpêtrière, Sorbonne Université, Paris, France

**Keywords:** Acute respiratory distress syndrome, Mechanical ventilation, COVID-19, Outcome, Predictive survival model

## Abstract

**Background:**

Predicting outcomes of critically ill intensive care unit (ICU) patients with coronavirus-19 disease (COVID-19) is a major challenge to avoid futile, and prolonged ICU stays.

**Methods:**

The objective was to develop predictive survival models for patients with COVID-19 after 1-to-2 weeks in ICU. Based on the COVID–ICU cohort, which prospectively collected characteristics, management, and outcomes of critically ill patients with COVID-19. Machine learning was used to develop dynamic, clinically useful models able to predict 90-day mortality using ICU data collected on day (D) 1, D7 or D14.

**Results:**

Survival of Severely Ill COVID (SOSIC)-1, SOSIC-7, and SOSIC-14 scores were constructed with 4244, 2877, and 1349 patients, respectively, randomly assigned to development or test datasets. The three models selected 15 ICU-entry variables recorded on D1, D7, or D14. Cardiovascular, renal, and pulmonary functions on prediction D7 or D14 were among the most heavily weighted inputs for both models. For the test dataset, SOSIC-7’s area under the ROC curve was slightly higher (0.80 [0.74–0.86]) than those for SOSIC-1 (0.76 [0.71–0.81]) and SOSIC-14 (0.76 [0.68–0.83]). Similarly, SOSIC-1 and SOSIC-7 had excellent calibration curves, with similar Brier scores for the three models.

**Conclusion:**

The SOSIC scores showed that entering 15 to 27 baseline and dynamic clinical parameters into an automatable XGBoost algorithm can potentially accurately predict the likely 90-day mortality post-ICU admission (sosic.shinyapps.io/shiny). Although external SOSIC-score validation is still needed, it is an additional tool to strengthen decisions about life-sustaining treatments and informing family members of likely prognosis.

**Supplementary Information:**

The online version contains supplementary material available at 10.1186/s13613-021-00956-9.

## Introduction

Since January 2020, the world has been massively affected by the coronavirus-19 disease (COVID-19) outbreak. In that context, intensive care units (ICUs) are frequently forced to expand bed capacity in many countries. Unusually long mechanical ventilation (MV) duration and ICU stays observed during the first wave are some of the most distinctive characteristics of treating severe acute respiratory syndrome-coronavirus 2 (SARS-CoV-2)-infection-related acute respiratory distress syndrome (ARDS), with 90-day mortality ranging from 31 to 53% [1–4]. Although accurately predicting patients’ clinical outcomes throughout this prolonged ICU stay can be difficult, effective recognition—at ICU admission and within the first 14 days—of those at high risk of death in-ICU is crucial to inform clinical decision-making and families of likely prognoses. It could also facilitate adequate resource allocation, including hospital beds and critical care resources, and risk-adjusted comparison of center-specific outcomes. Predicting outcomes of critically ill patients with COVID-19 being treated in the ICU is a major challenge, aimed at avoiding futile prolonged ICU stays and resource use, and provide additional reliable information for decision-making concerning withholding or withdrawing life-sustaining treatment, especially within disease epicenters needing to triage the high-volume influx of patients.

COVID-19-survival models published to date tried to predict the risk of clinical deterioration of acute cases [5, 6] using data from hospitalization day (D)1 [7]. To the best of our knowledge, none focused on predicting the survival of patients after 1-to-2 weeks in ICU. Taking advantage of the COVID–ICU-cohort database containing prospectively collected characteristics, management, and outcomes of patients admitted to ICUs for severe COVID-19 in France, Belgium, and Switzerland, between February and May 2020 [3], we used machine learning to develop three dynamic, clinically useful models able to predict 90-day mortality using in-ICU data collected on ICU D1, D7 or D14, respectively.

## Patients and methods

### Study population and data collection

COVID–ICU is a multicenter, prospective cohort study, conducted in 149 ICUs from 138 centers, across three countries (France, Switzerland, and Belgium), launched by the Reseau Europeen de recherche en Ventilation Artificielle (REVA) network. Most of the centers were in France (135/138) whereas two were in Belgium and one in Switzerland. All consecutive patients, over 16 years old, admitted to the participating ICUs between February 25, 2020, and May 4, 2020, with laboratory-confirmed SARS-CoV-2 infection, were included. Among the 4643 patients admitted to the ICU, 4244 had available survival status up to D90 post-ICU admission.

Every day, study investigators completed a standardized electronic case report form. Details of the information collected are described elsewhere [3]. Briefly, baseline information collected within the first 24 h post-ICU admission (D1) were: age, sex, body mass index (BMI), Simplified Acute Physiology Score (SAPS)-II [8], Sequential Organ-Failure Assessment (SOFA) score [9], comorbidities, clinical frailty-scale category [10], date of the first symptom(s) and ICU admission date. A daily-expanded dataset included respiratory support devices (oxygen mask, high-flow nasal cannula, noninvasive ventilation, or invasive MV), arterial blood gases, standard laboratory parameters, and adjuvant therapies for ARDS until D90. In-ICU organ dysfunctions included acute kidney failure requiring renal replacement therapy, proven thromboembolic complications, confirmed ventilator-associated pneumonia (VAP) or bacterial coinfection, and cardiac arrest. Each patient’s vital status was obtained 90 days post-ICU admission.

COVID–ICU received approval from the French Intensive Care Society Ethics Committee (CE-SRLF 20–23) in accordance with local regulations. All patients, or close relatives, were informed that their data were included in the COVID–ICU cohort. This study was conducted in accordance with the amended Declaration of Helsinki.

### Candidate predictors

We included candidate predictors considered in our previous multivariate Cox regression analyses, which assessed baseline risk factors of death by D90 [3]. D7 and D14 candidate predictors were defined a priori among data available in the COVID–ICU cohort [3] (i.e., before the building of the SOSIC models), based on recent publications describing risk factors and specific complications associated with COVID-19 prognosis [11, 12]. VAP was diagnosed by quantitative distal bronchoalveolar lavage cultures growing ≥ 10^4^ CFU/mL, blind protected specimen-brush distal samples growing ≥ 10^3^ CFU/mL, or endotracheal aspirates growing ≥ 10^6^ CFU/mL. Pulmonary embolism was proven by pulmonary computed-tomography angiography or echocardiography.

### Statistical analyses

#### Model development

We implemented a systematic machine learning-based framework to construct three mortality-prediction models (SOSIC-1, SOSIC-7, and SOSIC-14) from randomly selected development datasets, comprising 90% of the study sample; the remaining 10% were randomly assigned to the test datasets. Each prediction model was built using a gradient-boosting machine with decision trees, as implemented in the eXtreme Gradient-Boosting (XGBoost) classification algorithm [13]. XGBoost algorithm contains several tuning parameters (e.g., the number of decision trees, the maximal length of the component decision trees). The best set of parameters was chosen among a large grid of tuning parameters using tenfold cross-validation to maximize the prediction model’s discrimination ability, as assessed by the area under the receiver operating characteristics curve (AUC). We aimed to build models that could accurately estimate D90 survival for patients alive on D1, D7, or D14 following ICU admission. The SOSIC-1 model included only baseline candidate predictors, while SOSIC-7 and SOSIC-14 models combined baseline and D7 or D14 patient characteristics. The variable importance, which quantifies how much each variable contributed to the classification was extracted from the models. SHAP (SHapley Additive exPlanations) values were also computed to visualize the influence of each input variable on the final score [14].

#### Model validation

The performances of the three SOSIC models predicting 90-day mortality were evaluated using AUC-assessed discrimination (i.e., the probability that patients who experience the outcome will be ranked above those who do not), and calibration (i.e., the agreement between predicted and observed risks) assessed by the calibration curve (i.e., the ideal calibration intercept is 0 and ideal calibration slope is 1). The Brier score was also computed; it combines calibration and discrimination by quantifying how close predictions are to the observed outcomes (i.e., better performance is observed with a lower Brier score) [15].

A double internal validation was applied for the three SOSIC prediction models. First, internal validity was assessed by estimating the model performance corrected from optimism using bootstrap resampling with 100 repetitions. All the steps leading to the final prediction model (including the selection of the set of XGBoost tuning parameters) were applied to every bootstrap sample [16]. Second, model performance was assessed on the independent test datasets, distinct from development datasets used for model construction. One of the advantages of the XGBoost algorithm is its sparsity awareness that can handle the possibility of missing values [17]. Therefore, no missing value was imputed before model development or validation. Because the COVID-19 pandemic did not hit similarly all regions, we tested the performances of the three SOSIC models in two distinct populations namely in centers from Paris-greater areas and Grand Est compared to centers from other regions. Lastly, the performances of the SOSIC-1 were also compared to the SOFA and the SAPS II scores in the development and test datasets.

#### Descriptive analysis

Characteristics of the data included in the SOSIC scores are expressed as number (percentage) for categorical variables and means ± standard deviations or medians (interquartile ranges) for continuous variables. In a univariate analysis, categorical variables were compared with *χ*^2^ or Fisher’s exact test and continuous variables were compared with Student's *t*-test or Wilcoxon's rank-sum test. A *P* value < 0.05 was considered statistically significant. Statistical analyses and predictive model construction were computed with R v4.0.3, caret package v6.0-86, and XGBoost package v1.3.2.1.

## Results

### Study population

**Fig. 1:**
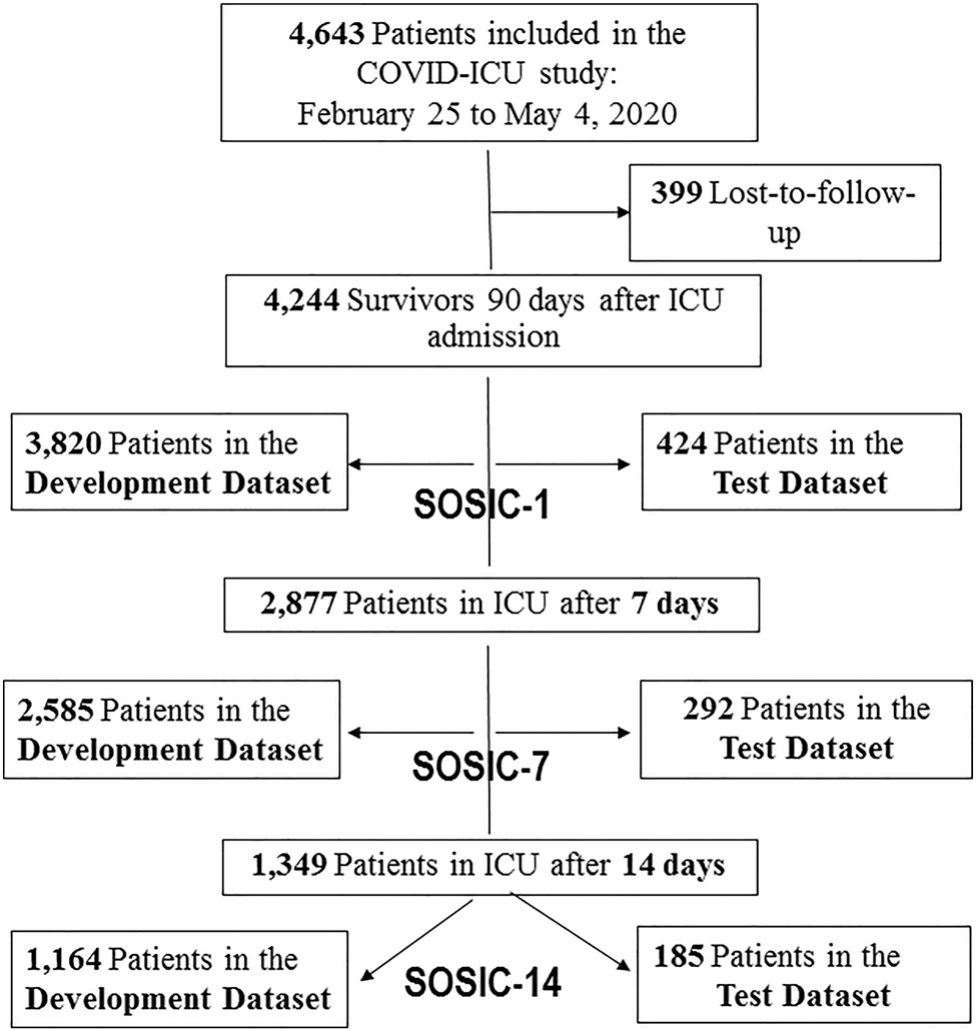
Flowchart of COVID-19 patient screening, inclusion, and assignment to the development and test datasets. *ICU* intensive care unit, *SOSIC* Survival of Severely Ill COVID score

Among 4643 patients enrolled by May 4, 2020, 399 were lost to follow-up by D90. Thus, the predictive survival models were built based on the remaining 4244 patients with available D90 vital status. Then, 4244, 2877, and 1349 patients, respectively, were included in the development datasets to construct the SOSIC-1, SOSIC-7, and SOSIC-14 scores, with 424, 292, and 185 from each group, respectively, randomly assigned to the corresponding test datasets (Fig. 1). The three models selected 15 ICU (baseline) variables: i.e., age; sex; BMI; treated hypertension; known diabetes; immunocompromised status; clinical frailty-scale category; bacterial coinfection; ventilation profile; SOFA-score respiratory, cardiovascular, and renal components; lactate concentration; and lymphocyte count). d-Dimers were also selected a priori but were not retained for model development because of their inconsistent collection at ICU admission (Additional file 1). Selected in-ICU parameters obtained on D7 (SOSIC-7) or D14 (SOSIC-14) were: SOFA-score respiratory, cardiovascular, and renal components; lactate level, and ventilation profile. In addition, on D7 or D14, the duration of invasive MV, extubation procedure, prone-positioning, continuous neuromuscular blockade, VAP, cardiac arrest, and/or proven pulmonary embolism since ICU admission were integrated into the SOSIC-7 and SOSIC-14 scores. Table 1 reports the distributions of these variables according to D90 vital status in the D1, D7, or D14 development and test datasets.

**Table 1:** Demographic, clinical and ventilatory support characteristics of the development and test datasets of the SOSIC scores on ICU day-1 according to their 90-day survival status

	SOSIC-1						SOSIC-7						SOSIC-14					
	Development dataset (*n* = 3820)			Test dataset (*n* = 424)			Development dataset (*n* = 2585)			Test dataset (*n* = 292)			Development dataset (*n* = 1164)			Test dataset (*n* = 185)		
	90-day status			90-day status			90-day status			90-day status			90-day status			90-day status		
	Alive (2665)	Died (1165)	*P* value	Alive (291)	Death (133)	*P* value	Alive (1845)	Died (740)	*P* value	Alive (217)	Died (75)	*P* value	Alive (1251)	Died (413)	*P* value	Alive (142)	Died (43)	*P* value
Age, years	61 (53–69)	68 (59–74)	< 0.01	61 (50–68)	65 (58–73)	< 0.01	61 (53–69)	67 (59–73)	< 0.01	61 (51–68)	64 (58–72)	0.02	62 (53–69)	66 (58–72)	< 0.01	63 (52–69)	64 (56–72)	0.20
Women	696 (26)	286 (25)	0.30	75 (26)	28 (21)	0.30	467 (25)	169 (23)	0.20	54 (25)	11 (15)	0.07	299 (24)	84 (20)	0.82	34 (24)	4 (9)	0.04
Body mass index, kg/m^2^	28 (26–32)	28 (25–32)	0.01	29 (26–33)	28 (25–31)	0.20	29 (26–33)	28 (25–32)	< 0.01	29 (26–33)	29 (26–31)	0.80	29 (26–33)	28 (25–31)	< 0.01	29 (25–34)	29 (25–30)	0.30
Treated hypertension	1,173 (45)	637 (55)	< 0.01	137 (47)	71 (54)	0.20	848 (46)	388 (53)	0.03	101 (47)	38 (51)	0.50	593 (48)	204 (50)	0.50	72 (51)	20 (48)	0.70
Known diabetes	632 (24)	416 (36)	< 0.01	72 (25)	47 (36)	0.02	449 (25)	261 (36)	< 0.01	51 (24)	25 (34)	0.08	313 (25)	141 (35)	< 0.01	35 (25)	12 (29)	0.60
Immunocompromised^a^	162 (6)	121 (11)	< 0.01	16 (6)	15 (12)	0.03	123 (7)	67 (9)	0.03	14 (6)	8 (11)	0.20	88 (7)	37 (9)	0.2	8 (6)	1 (2)	0.70
Clinical frailty scale			< 0.01			< 0.01			< 0.01			0.30			< 0.01			0.70
1–2	1611 (67)	466 (45)		188 (58)	57 (47)		1100 (66)	334 (51)		143 (71)	37 (55)		736 (66)	205 (56)		91 (59)	24 (61)	
3–4	725 (30)	459 (39)		76 (27)	51 (42)		515 (31)	174 (42)		52 (25)	27 (40)		398 (32)	151 (41)		38 (29)	14 (36)	
≥ 5	70 (3)	104 (9)		10 (4)	12 (10)		42 (3)	46 (7)		8 (4)	3 (5)		29 (3)	14 (4)		3 (2)	1 (3)	
1st symptom(s)-to-ICU admission interval, day	9 (7–12)	8 (5–11)	< 0.01	9 (7–12)	8 (5–11)	< 0.01	9 (7–11)	8 (5–11)	< 0.01	9 (7–12)	8 (5–10)	0.02	9 (6–11)	8 (5–11)	0.20	10 (7–12)	8 (5–10)	0.01
**1st 24 h in ICU**																		
Bacterial coinfection	173 (7)	95 (8)	0.06	15 (5)	15 (12)	0.02	130 (7)	59 (8)	0.40	12 (9)	4 (9)	0.90	86 (7)	35 (9)	0.81	12 (9)	4 (9)	0.90
Ventilation profile^b^			< 0.01			0.01			< 0.01			0.70			< 0.01			0.40
Standard O_2_ therapy	575 (22)	115 (10)		61 (21)	15 (12)		269 (15)	73 (10)		31 (14)	9 (12)		152 (12)	41 (10)		17 (12)	6 (14)	
High-flow O_2_	403 (16)	105 (9)		46 (16)	13 (10)		229 (13)	57 (8)		29 (14)	8 (11)		114 (9)	30 (7)		11 (8)	4 (9)	
Noninvasive ventilation	88 (3)	57 (5)		7 (2)	6 (5)		44 (2)	36 (5)		5 (2)	3 (4)		27 (2)	21 (5)		2 (1)	2 (5)	
Invasive MV	1505 (59)	861 (76)		173 (60)	96 (74)		1259 (70)	564 (77)		54 (73)	149 (70)		928 (76)	315 (77)		30 (71)	112 (79)	
SOFA components																		
Respiratory	3 (2–3)	3 (2–4)	< 0.01	3 (2–3)	3 (2.7–4)	< 0.01	3 (2–3)	3 (3–4)	< 0.01	3 (2–3)	3 (2–4)	0.40	3 (2–3)	3 (3–4)	0.02	3 (3–4)	3 (2–4)	0.90
Cardiovascular	0 (0–3)	3 (0–4)	< 0.01	0 (0–3)	1 (0–4)	0.05	1 (0–4)	3 (0–4)	< 0.01	1 (0–3)	0 (0–3)	0.90	1 (0–4)	3 (0–4)	0.01	1 (0–3)	0 (0–3)	0.93
Renal	0 (0–0)	0 (0–1)	< 0.01	0 (0–0)	1 (0–2)	< 0.01	0 (0–0)	0 (0–1)	< 0.01	0 (0–0)	0 (0–2)	< 0.01	0 (0–0)	0 (0–1)	< 0.01	0 (0–0)	0 (0–1)	0.01
Lactate, mmol/L	1.2 (0.9–1.5)	1.3 (1.0–1.8)	< 0.01	1.2 (0.9–1.5)	1.3 (1.0–1.7)	< 0.01	1.2 (0.9–1.5)	1.3 (1.0–1.8)	< 0.01	1.2 (0.9–1.5)	1.3 (1.0–1.7)	0.20	1.2 (1.0–1.6)	1.4 (1.0–1.8)	< 0.01	1.2 (1.0–1.6)	1.1 (0.9–1.4)	0.10
Lymphocytes, × 10^9^/L	0.8 (0.6–1.2)	0.8 (0.5–1.1)	< 0.01	0.8 (0.6–1.1)	0.7 (0.5–1.2)	0.70	0.8 (0.5–1.1)	0.7 (0.5–1.1)	< 0.01	0.8 (0.6–1.1)	0.7 (0.5–1.3)	0.90	0.8 (0.5–1.1)	0.7 (0.5–1.1)	0.03	0.8 (0.5–1.1)	0.7 (0.5–1.2)	0.60

Results are expressed as n (%) or median (25th–75th percentiles)

*SOFA* Sequential Organ-Failure Assessment, *MV* mechanical ventilation

^a^Defined as hematological malignancies, active solid tumor, or having received specific anti-tumor treatment within a year, solid-organ transplant, human immunodeficiency virus-positive, or immunosuppressants

^b^Only the most intensive ventilation modality (i.e., invasive MV > noninvasive ventilation > high-flow oxygen > standard oxygen) during the first 24 h is reported

Univariate analyses of patient characteristics in the development datasets showed that those who died were significantly older and had a higher clinical frailty-scale category, lower BMI, and shorter intervals between first symptom(s) and ICU admission (except for the SOSIC-14 dataset) compared to D90 survivors (*P* < 0.01). Similarly, patients who had died by D90 were more likely on invasive MV in ICU D1 and had significantly higher SOFA-score respiratory, cardiovascular, and renal components. Their lactate levels during the first 24 h in-ICU were significantly higher and lymphocyte counts were lower.

Among patients still in-ICU on D7 or D14, the same differences were observed regarding their SOFA-score components, lactate levels, and ventilation profiles on those days (Table 2). As expected, patients who died were more likely to have undergone prone-positioning or received neuromuscular blockade and experienced significantly more complications in-ICU (i.e., VAP, cardiac arrest, pulmonary embolism) within the first 7 or 14 days.

**Table 2:** Characteristics on ICU day-7 or day-14 of the development and test datasets of the SOSIC-7 and SOSIC-14 scores according to their 90-day survival status

	SOSIC-7						SOSIC-14					
	Development dataset (*n* = 2585)			Test dataset (*n* = 292)			Development dataset (*n* = 1164)			Test dataset (*n* = 185)		
	90-day status			90-day status			90-day status			90-day status		
	Alive (1845)	Death (740)	*P* value	Alive (217)	Death (75)	*P* value	Alive (1251)	Death (413)	*P* value	Alive (142)	Death (43)	*P* value
On day-7 or day-14 in ICU												
SOFA components												
Respiratory	3 (2–3)	3 (3–4)	< 0.01	3 (2–3)	3 (3–4)	< 0.01	3 (2–3)	3 (3–4)	< 0.01	3 (2–3)	3 (3–4)	0.02
Cardiovascular	3 (1–4)	4 (3–4)	< 0.01	3 (1–4)	4 (3–4)	< 0.01	3 (1–4)	4 (3–4)	< 0.01	3 (1–4)	4 (3–4)	0.10
Renal	2 (1–4)	2 (1–4)	0.20	2 (1–4)	2 (2–4)	0.5	2 (1–4)	2 (1–4)	0.3	2 (1–4)	3 (2–4)	0.20
Lactate, mmol/L	1.3 (1.0–1.7)	1.5 (1.2–1.9)	< 0.01	1.3 (1.0–1.7)	1.4 (1.1–1.8)	0.06	1.1 (0.8–1.5)	1.3 (1.0–1.7)	< 0.01	1.2 (0.9–1.6)	1.2 (0.9–1.5)	0.60
Ventilation profile^a^			< 0.01			0.30			< 0.01			0.02
Standard oxygen therapy	96 (5)	4 (0.5)		11 (5)	1 (1)		96 (8)	0 (0)		17 (12)	0 (0)	
High-flow oxygen	98 (5)	7 (1)		15 (7)	2 (3)		40 (3)	4 (1)		6 (4)	0 (0)	
Noninvasive ventilation	37 (2)	10 (1)		5 (2)	1 (1)		32 (3)	3 (1)		4 (3)	1 (2)	
Invasive MV	1578 (87)	714 (97)		186 (86)	71 (95)		1040 (86)	401 (98)		110 (80)	42 (98)	
**Since ICU admission**												
Invasive MV duration	7 (6–7)	7 (7–7)	< 0.01	7 (6–7)	7 (6–7)	0.40	14 (13–14)	14 (13–14)	< 0.01	14 (13–14)	14 (12–14)	0.80
Extubation	76 (5)	8 (1)	< 0.01	8 (4)	2 (3)	0.90	153 (13)	12 (3)	< 0.01	19 (14)	4 (9)	0.50
≥ 1 prone-positioning session	1028 (63)	531 (75)	< 0.01	127 (67)	58 (82)	0.02	923 (76)	346 (86)	< 0.01	105 (77)	35 (83)	0.40
≥ 24 h continuous neuromuscular blockades	1408 (86)	653 (92)	< 0.01	151 (79)	61 (86)	0.20	1095 (91)	384 (96)	< 0.01	122 (90)	40 (95)	0.40
≥ 1 VAP episode(s)	597 (36)	276 (41)	0.02	57 (28)	21 (30)	0.70	738 (62)	266 (69)	0.01	82 (59)	21 (50)	0.30
Cardiac arrest	26 (1)	18 (2)	0.06	2 (1)	3 (4)	0.11	44 (3)	18 (4)	0.40	2 (1)	3 (7)	0.08
Proven pulmonary embolism	74 (4)	39 (5)	0.15	10 (5)	4 (5)	0.80	102 (8)	42 (10)	0.20	15 (11)	6 (14)	0.60

Results are expressed as n (%) or median (25th–75th percentiles). *ICU* intensive care unit, *SOFA* Sequential Organ-Failure Assessment, *MV* mechanical ventilation, *VAP* ventilator-associated pneumonia

^a^Only the most intensive ventilation modality (i.e., invasive MV > noninvasive ventilation > high-flow oxygen > standard oxygen) on day-7 or day-14 is reported

### Importance of the 90-day mortality predictors

**Fig. 2:**
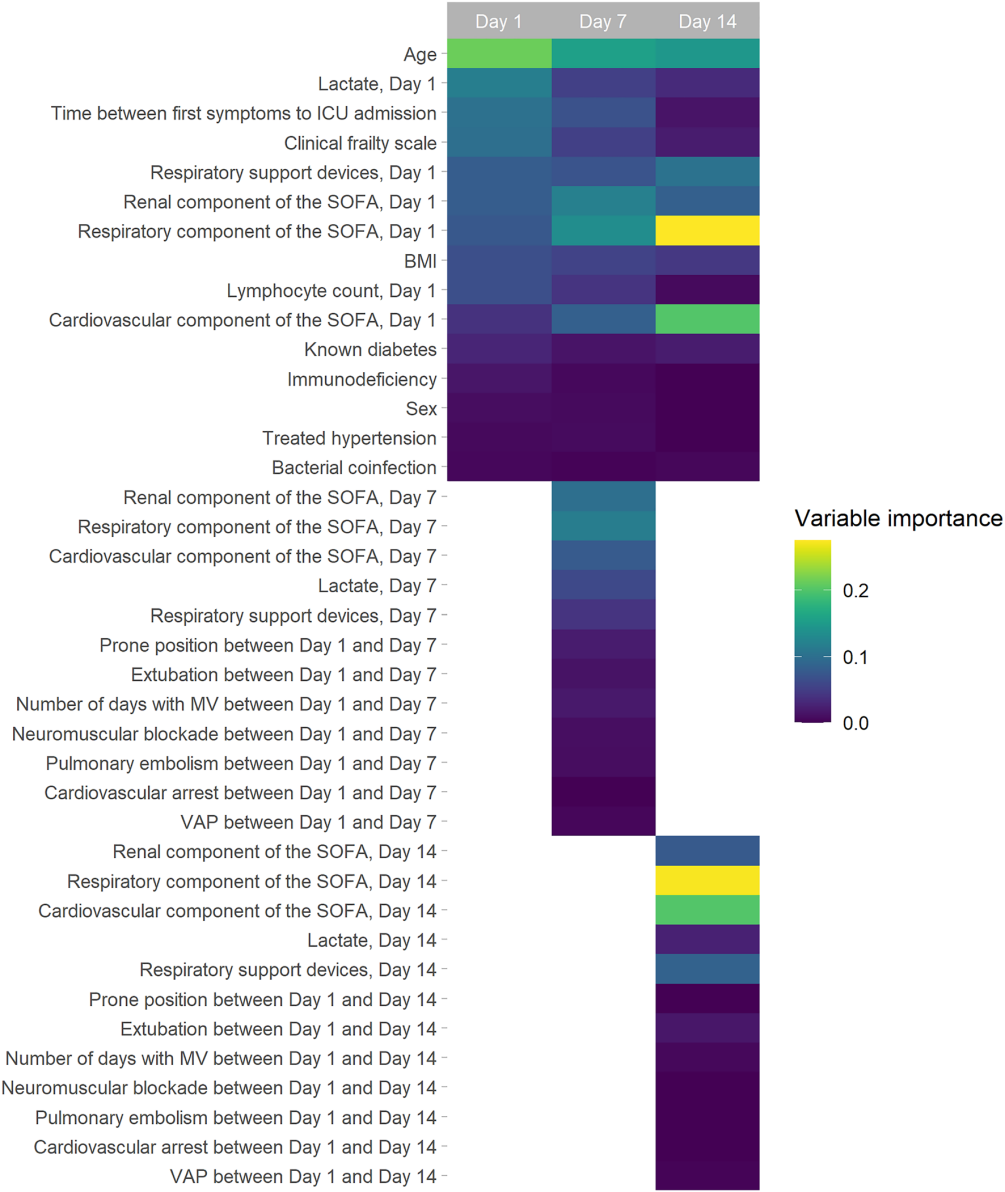
Variable weighting in the machine-learning models: Survival of Severely Ill COVID (SOSIC) scores on days 1, 7 and 14. A color gradient is used to show variable strength in the machine-learning models at different times, ranging from yellow for the highest preponderance input variables to progressively darker shade of purple for lower input variables. *D* day, *BMI* body mass index, *ICU* intensive care unit, *MV* mechanical ventilation, *SOSIC* Survival of Severely Ill COVID score, *SOFA* Sequential Organ-Failure Assessment, *VAP* ventilator-associated pneumonia

Figure 2 and Additional file 2 illustrate variable-weighting in the machine-learning models used to build the D1, D7, and D14 SOSIC scores. Briefly, age, clinical frailty-scale category, D1 lymphocyte count, and the interval between first symptom(s) and ICU admission were given significant weight to predict D90 mortality. However, the weights of these baseline characteristics tended to decrease when the prediction was estimated after 7 or 14 days in-ICU. Conversely, other baseline comorbidities, such as known diabetes, immunocompromised status, or treated hypertension, were accorded similar weights in all three scores.

Interestingly, when the prediction was estimated on D7 (SOSIC-7) or D14 (SOSIC-14), SOFA-score respiratory and cardiovascular components, and respiratory support at ICU admission were accorded greater importance compared to the D1 prediction. Moreover, cardiovascular, renal, and pulmonary functions on the prediction D7 or D14 were among the inputs with the highest preponderance in both models, while in-ICU complications since admission had only modest weight.

### Performance of the SOSIC scores

**Fig. 3:**
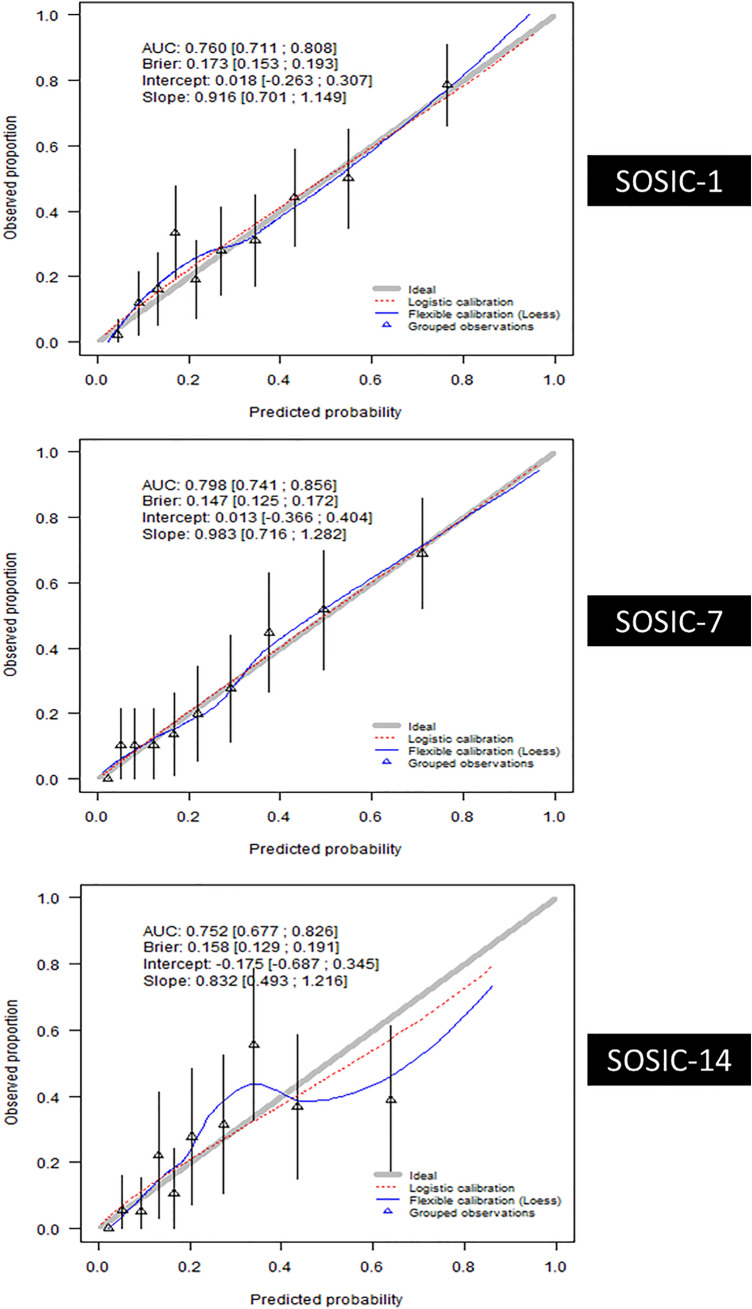
Calibration and discrimination of the Survival of Severely Ill COVID (SOSIC)-1, SOSIC-7 and SOSIC-14 scores using the test dataset

**Fig. 4:**
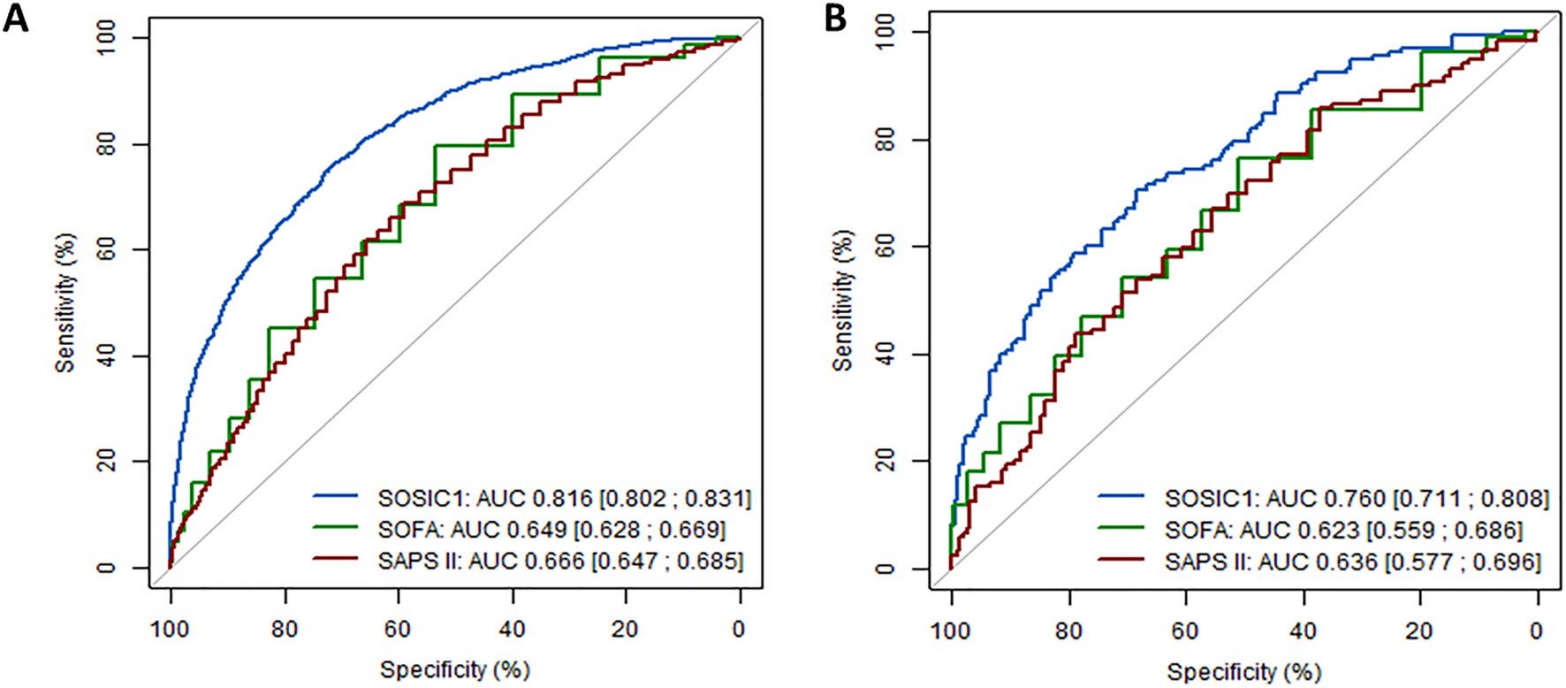
Graphic representation of the SOSIC-1, the SAPS II, and the SOFA performances in the **A** development and **B** the test datasets. *SOSIC* Survival of Severely Ill COVID score; *SAPS II* Simplified Acute Physiology Score II, *SOFA* Sequential Organ-Failure Assessment

We developed three models using XGBoost algorithms that accurately predicted 90-day mortality using data from ICU D1 and the prediction day. Apparent-, bootstrap-corrected-, and test-dataset-validation metrics are reported in the Additional file 3. Based on the test dataset the AUC was slightly higher for SOSIC-7 (0.80 [0.74–0.86]) than for SOSIC-1 (0.76 [0.71–0.81]) and SOSIC-14 (0.76 [0.68–0.83]). Similarly, SOSIC-1 and SOSIC-7 calibration curves were excellent **(**Fig. 3). Those findings indicate fair agreement between predicted and observed risks in those two scores. Although calibration of SOSIC-14 was lower (slope 0.83 [0.49–1.22]) compared to the SOSIC-1 and SOSIC-7, the Brier scores, which assess both calibration and discrimination of the predictive models, were similar for all three models. Besides, the correlations between the three scores were good (Additional file 4). We did not identify any center effect, as evidenced by the AUC of SOSIC-1 which was similar in Paris-greater areas and Grand Est vs other regions (0.75 95%CI [0.69;0.81] vs 0.76 95%CI [0.67;0.85]). Similarly, calibration slope close to one whatever the region (Paris-greater area and Grand Est AUC: 0.92 95%CI [0.65;1.21] versus other regions AUC: 0.90 95%CI [0.53;1.33]). Similar results were observed for SOSIC-7 and SOSIC-14 models and are reported in Additional file 5. The internal validation of SOSIC-1 on the test dataset exhibited fair performance (AUC: 0.76 [95%CI 0.71;0.81]) in contrast to much poorer discrimination of the SAPS II (AUC:0.64 [95%CI 0.58;0.70]) and SOFA scores (AUC:0.62 [95%CI 0.56; 0.69]). Graphic representation of the SOSIC-1, SAPS II, and SOFA discrimination performances is shown in Fig. 4. Similarly, performances of the SOSIC-7 and SOSIC-14 were slightly better than SOFA at day-7 and day-14, respectively (Additional file 6).

## Discussion

We developed and validated three prognostic models (SOSIC-1, SOSIC-7, and SOSIC-14) to predict 90-day mortality of 4244 critically ill patients with COVID-19 treated in France, Belgium, and Switzerland, evaluated during the 2 weeks following ICU admission. The SOSIC scores showed that entering 15 to 27 baseline and dynamic clinical parameters (depending on the score day) into an automatable XGBoost algorithm had the potential to accurately predict likely mortality 90 days post-ICU admission. Although external validations of the SOSIC scores in other critically ill populations with COVID-19 are still needed, these dynamic tools could enable clinicians to objectively assess the in-ICU mortality risk of patients with COVID-19 for up to 14 days. It offers an additional tool to strengthen decisions about life-sustaining treatments, hospital and ICU resources, and informing family members of likely prognosis.

Predicting outcomes of critically ill COVID patients is challenging. Patients hospitalized with COVID-19 can be classified into three phenotypes that have prognostic implications [18]. Indeed, patients with more chronic heart, lung, or renal disease(s), obesity, diabetes, an intense inflammatory syndrome, higher creatinine level, and poorer oxygenation parameters were classified as having the highest risk of deterioration that was associated with poorer outcomes [18]. Age is frequently associated with higher rates of hospitalization, ICU admission, and mortality of patients with COVID-19 [18–20]. Frailty is a useful tool to stratify the risk of death 90 days post-ICU admission and offers important additional prognostic information to combine with age over 70 years for patients with COVID [21]. Interestingly, the weights accorded age and frailty in our predictive models declined over the ICU stay. In other words, those two variables more weakly affected mortality prediction after 7 or 14 days in-ICU, compared to the prediction at ICU admission (Fig. 2). Similarly, a shorter interval from the onset of COVID-19 symptom(s) to ICU admission, which was associated with a higher risk of death [22], weighed less in SOSIC-7 and SOSIC-14.

Despite being collected on D1, SOFA-score cardiovascular, respiratory, and renal components strongly impacted later predictions but only modestly affected the D1 prediction. Similarly, in an observational multicenter cohort of patients with moderate to severe COVID-19 ARDS, the decrease of the static compliance of the respiratory system observed between ICU day-1 and day-14 was not associated with day-28 outcome [23]. Besides, cardiac injuries appear frequent with nearly 70% of COVID-19 patients experienced cardiac injury within the first 14 days of ICU stay [24].

The poor discriminant accuracy of the SOFA-score to predict mortality of patients before intubation for COVID-19 pneumonia was recently highlighted [25]; indeed, these patients generally have severe single-organ dysfunction and globally less SOFA-score variations. However, the impacts D7 and D14 respiratory, cardiovascular and renal statuses are of the utmost importance in the mortality prediction at those times. The SOSIC scores put the spotlight on the possibility of some variables exerting variable influence to predict mortality of patients with COVID-19, e.g., demographic variables had less weight after 1 or 2 weeks in-ICU. However, the discrimination of the SOSIC scores did not improve over time. Indeed, the AUC was not better at day-7 or 14 compared to day-1 and its better performance compared to the SOFA-score was reduced over time. A greater number of variables inducing a higher heterogeneity associated with a reduction of the sample size of the development and the test datasets at day-7 and 14 could explain this finding.

Because no models predicting COVID-19 outcomes focused on patients already in the ICU [5, 6, 26], the SOSIC scores have the potential for clinical usefulness and generalizability. Internal validations of the SOSIC scores showed consistent discrimination and calibration, which obviously deserves further external validation. With an AUC around 0.80, external validation is desirable to assess the mortality prediction beyond population levels and to fully assess the mortality risk of the individual being admitted and cared for up to 2 weeks in-ICU. Although discrimination was largely consistent for the different validation methods, SOSIC-14’s calibrations were lower; that finding suggests its performance using an external independent sample might be lower than those of SOCIC-1 or SOSIC-7. By construction, SOSIC-14 was developed on a smaller sample size than the other two models, which might explain its lower quality in terms of predicting 90-day mortality.

Despite being developed and validated on a substantial cohort with a large number of participating ICUs, these scores were constructed during the first COVID wave in Europe, a period with high pressure on the health systems and before the publication of core randomized trials [4, 27]. Moreover, ventilator strategies have also changed, as the pandemic has evolved and the medical community acquired a greater understanding of the pathophysiology of the disease and how to treat it. Caregiver reluctance to provide noninvasive oxygen strategies has been overcome [3], leading to higher percentages of patients on high-flow oxygen and noninvasive ventilation, and lower rates of intubation on ICU D1 [3, 28].

Debates are still ongoing as to the best timing of intubation in that population, as recent data have suggested poorer outcomes associated with an early intubation strategy [29–31]. Thus, the very high percentages of our patients intubated on ICU D1 will probably differ during subsequent COVID-19 outbreaks, in countries with different public healthcare organizations or ICU admission policies.

Indeed, SOSIC predictions should be interpreted as reflecting a profile of critically ill patients with COVID-19 not routinely treated with corticosteroids and outside vaccination campaigns, which may have changed since May 2020. Besides, this cohort was conducted at a time where the national health system was extremely pressured which lead to an important reorganization of intensive care supplies in some regions although we did not find a region effect on the performances of the SOSIC scores. However, we cannot rule out that outside a surge situation, the model could slightly overestimate the mortality. As commonly done for other scoring systems [32], prospective external validations of the SOSIC scores are warranted to determine the need for temporal recalibration and to evaluate model performance in diverse international settings. External validations in more recent cohorts of patients who received recent treatments and ventilation management are warranted. The publicly available calculator (sosic.shinyapps.io/shiny) should help achieve these goals. Another limitation is that we only included predictors that were routinely collected in the COVID–ICU database during the study period. Thus, we cannot rule out that some additional laboratory or ventilatory parameters reflecting respiratory mechanics (especially measured on ICU D7 or D14) would have improved SOSIC-score performances. We were also unable to integrate d-dimer concentrations as initially planned [33], because of their inconsistent collection at ICU admission. Although the XGBoost algorithm incorporates missing data in its split finding algorithm, we cannot guarantee that this method can handle any pattern of missing data effectively [34]. As this algorithm potentially exploits the data missingness patterns for prediction, a major shift in the missingness mechanism in an external independent sample may affect SOSIC scores performance. Lastly, important detailed information on therapy withholding or withdrawing is lacking.

## Conclusion

The SOSIC-1, -7, and -14 scores were able to fairly predict 90-day mortality of critically ill patients with COVID-19 admitted and managed in-ICU (sosic.shinyapps.io/shiny). These machine-learning models, built with XGBoost algorithms, showed good discriminations and excellent calibrations. The patient’s demographic characteristics contributed most to SOSIC-1, while ventilatory status and extrapulmonary dysfunctions were the preponderant predictors in SOSIC-7 and SOSIC-14. Further studies are now warranted to externally validate these scores in recent cohorts of critically ill COVID-19 patients and assess their performances at individual levels as the pandemic evolves.

## Supplementary Information

**Additional file 1.** Number and percentage of missing variables in the SOSIC-1, SOSIC-7, and SOSIC-14 scores, respectively.

**Additional file 2.** SHAP (SHapley Additive exPlanations) values to visualize the influence of each input variable on the final score.

**Additional file 3.** Validation Metrics of the SOSIC-1, SOSIC-7, and SOSIC-14 Scores.

**Additional file 4.** Correlation between SOSIC-1, SOSIC-7, and SOSIC-14.

**Additional file 5.** Calibration and discrimination of the Survival of Severely Ill COVID (SOSIC)-1, SOSIC-7, and SOSIC-14 scores in centers from Paris-greater area and Grand Est vs other regions, respectively.

**Additional file 6.** Graphic representation of the performances of A) SOSIC-7 and the SOFA at day-7, and B) SOSIC-14 and the SOFA at day-14 in the development and the test datasets.

## Data Availability

Not applicable.

## Abbreviations

ARDS: Acute respiratory distress syndrome
AUC: Area under the receiver operating characteristics (ROC) curve
BMI: Body mass index
COVID-19: Coronavirus-19 disease
ICU: Intensive care units
MV: Mechanical ventilation
ML: Machine learning
SAPS II: Simplified Acute Physiology Score II
SARS-CoV-2: Severe acute respiratory syndrome-coronavirus-2
SOFA: Sequential Organ-Failure Assessment
SOSIC: Survival of Severely Ill COVID
VAP: Ventilator-associated pneumonia
