# Supplementary material for: Predicting 90-day survival of patients with COVID-19: Survival of Severely Ill COVID (SOSIC) scores
Source: Ann Intensive Care. 2021 Dec 11;11:170. doi: 10.1186/s13613-021-00956-9 (PMC8665857; doi:10.1186/s13613-021-00956-9)
Supplement: Supplementary file 1 — Additional file 1. Number and percentage of missing variables in the SOSIC-1, SOSIC-7, and SOSIC-14 scores, respectively. [file 13613_2021_956_MOESM1_ESM.docx]

**Additional file 1: Number and percentage of missing variables in the SOSIC-1, SOSIC-7, and SOSIC-14 scores, respectively.**

| **SOSIC-1** | **N** | **N missing** | **Percent missing** |
| --- | --- | --- | --- |
| Vital status | 4,244 | 0 | 0.0 |
| Age | 4,244 | 0 | 0.0 |
| Sex | 4,226 | 18 | 0.4 |
| BMI | 3,935 | 309 | 7.3 |
| Treated hypertension | 4,197 | 47 | 1.1 |
| Known diabetes | 4,196 | 48 | 1.1 |
| Immunodeficiency | 4,192 | 52 | 1.2 |
| Frailty score | 3,839 | 405 | 9.5 |
| Delay between first signs and ICU admission | 4,007 | 237 | 5.6 |
| Bacterial coinfection | 4,116 | 128 | 3.0 |
| Lactate, mmol/L | 3,792 | 452 | 10.7 |
| Lymphocyte count | 3,48 | 764 | 18.0 |
| Ventilation profil at admission | 4,126 | 118 | 2.8 |
| SOFA Respiratory system | 3,913 | 331 | 7.8 |
| SOFA Cardiovascular system | 4,065 | 179 | 4.2 |
| SOFA Renal system | 4,014 | 230 | 5.4 |

| **SOSIC-7** | **N** | **N missing** | **Percent missing** |
| --- | --- | --- | --- |
| Vital status | 2,877 | 0 | 0.0 |
| Age | 2,877 | 0 | 0.0 |
| Sex | 2,864 | 13 | 0.5 |
| BMI | 2,719 | 158 | 5.5 |
| Treated hypertension | 2,849 | 28 | 1.0 |
| Known diabetes | 2,845 | 32 | 1.1 |
| Immunodeficiency | 2,849 | 28 | 1.0 |
| Frailty score | 2,581 | 296 | 10.3 |
| Delay between first signs and ICU admission | 2,733 | 144 | 5.0 |
| Bacterial coinfection | 2,799 | 78 | 2.7 |
| Lactate | 2,603 | 274 | 9.5 |
| Lymphocyte count | 2,381 | 496 | 17.2 |
| Mechanical ventilation duration, Day 1 - Day 7 | 2,863 | 14 | 0.5 |
| Lactates, Day 7 | 2,465 | 412 | 14.3 |
| Extubation before Day 7 | 2,613 | 264 | 9.2 |
| VAP before Day 7 | 2,613 | 264 | 9.2 |
| Pulmonary embolism before Day 7 | 2,859 | 18 | 0.6 |
| Cardiac arrest before Day 7 | 2,86 | 17 | 0.6 |
| Prone position before Day 7 | 2,61 | 267 | 9.3 |
| Neuromuscular blockade before Day 7 | 2,608 | 269 | 9.4 |
| Respiratory support devices, Day 1 | 2,819 | 58 | 2.0 |
| SOFA Respiratory system, Day 1 | 2,695 | 182 | 6.3 |
| SOFA Cardiovascular system, Day 1 | 2,781 | 96 | 3.3 |
| SOFA Renal system, Day 1 | 2,738 | 139 | 4.8 |
| Respiratory support devices, Day 7 | 2,836 | 41 | 1.4 |
| SOFA Respiratory system, Day 7 | 2,682 | 195 | 6.8 |
| SOFA Cardiovascular system, Day 7 | 2,739 | 138 | 4.8 |
| SOFA Renal system, Day 7 | 2,709 | 168 | 5.8 |

| **SOSIC-14** | **N** | **N missing** | **Percent missing** |
| --- | --- | --- | --- |
| Vital status | 1,849 | 0 | 0.0 |
| Age | 1,849 | 0 | 0.0 |
| Sex | 1,84 | 9 | 0.5 |
| BMI | 1,755 | 94 | 5.1 |
| Treated hypertension | 1,827 | 22 | 1.2 |
| Known diabetes | 1,829 | 20 | 1.1 |
| Immunodeficiency | 1,834 | 15 | 0.8 |
| Frailty score | 1,666 | 183 | 9.9 |
| Delay between first signs and ICU admission | 1,752 | 97 | 5.2 |
| Bacterial coinfection | 1,805 | 44 | 2.4 |
| Lactate | 1,672 | 177 | 9.6 |
| Lymphocyte count | 1,55 | 299 | 16.2 |
| Mechanical ventilation duration, Day 1 - Day 14 | 1,842 | 7 | 0.4 |
| Lactates, Day 14 | 1,521 | 328 | 17.7 |
| Extubation before Day 14 | 1,791 | 58 | 3.1 |
| VAP before Day 14 | 1,76 | 89 | 4.8 |
| Pulmonary embolism before Day 14 | 1,838 | 11 | 0.6 |
| Cardiac arrest before Day 14 | 1,839 | 10 | 0.5 |
| Prone position before Day 14 | 1,789 | 60 | 3.2 |
| Neuromuscular blockade before Day 14 | 1,783 | 66 | 3.6 |
| Respiratory support devices, Day 1 | 1,812 | 37 | 2.0 |
| SOFA Respiratory system, Day 1 | 1,751 | 98 | 5.3 |
| SOFA Cardiovascular system, Day 1 | 1,791 | 58 | 3.1 |
| SOFA Renal system, Day 1 | 1,771 | 78 | 4.2 |
| Respiratory support devices, Day 14 | 1,796 | 53 | 2.9 |
| SOFA Respiratory system, Day 14 | 1,697 | 152 | 8.2 |
| SOFA Cardiovascular system, Day 14 | 1,758 | 91 | 4.9 |
| SOFA Renal system, Day 14 | 1,734 | 115 | 6.2 |
