# Supplementary material for: Predicting 90-day survival of patients with COVID-19: Survival of Severely Ill COVID (SOSIC) scores
Source: Ann Intensive Care. 2021 Dec 11;11:170. doi: 10.1186/s13613-021-00956-9 (PMC8665857; doi:10.1186/s13613-021-00956-9)
Supplement: Supplementary file 2 — Additional file 2. SHAP (SHapley Additive exPlanations) values to visualize the influence of each input variable on the final score. [file 13613_2021_956_MOESM2_ESM.docx]

**Additional file 2: SHAP (SHapley Additive exPlanations) values to visualize the influence of each input variable on the final score**

*The y-axis indicates the variable name, in order of importance from top to bottom. Variables were ordered according to the mean SHAP value, which is indicated next to the variable name. The x-axis indicates the SHAP value, which quantifies the difference between the prediction of the model (without the use of the considered variable) and the prediction of the model with the considered variable (in log-odds scale). Each point represents an individual from the dataset. Gradient color indicates the original value for one variable (for binary variables, it takes two colors).*

*Example of interpretation for the age variable: lower age is associated with a high decrease in SHAP value. Inversely, higher age is associated with a high increase in SHAP value. It means that higher age is associated with a higher risk of death.*

***SOSIC-1 :***

***
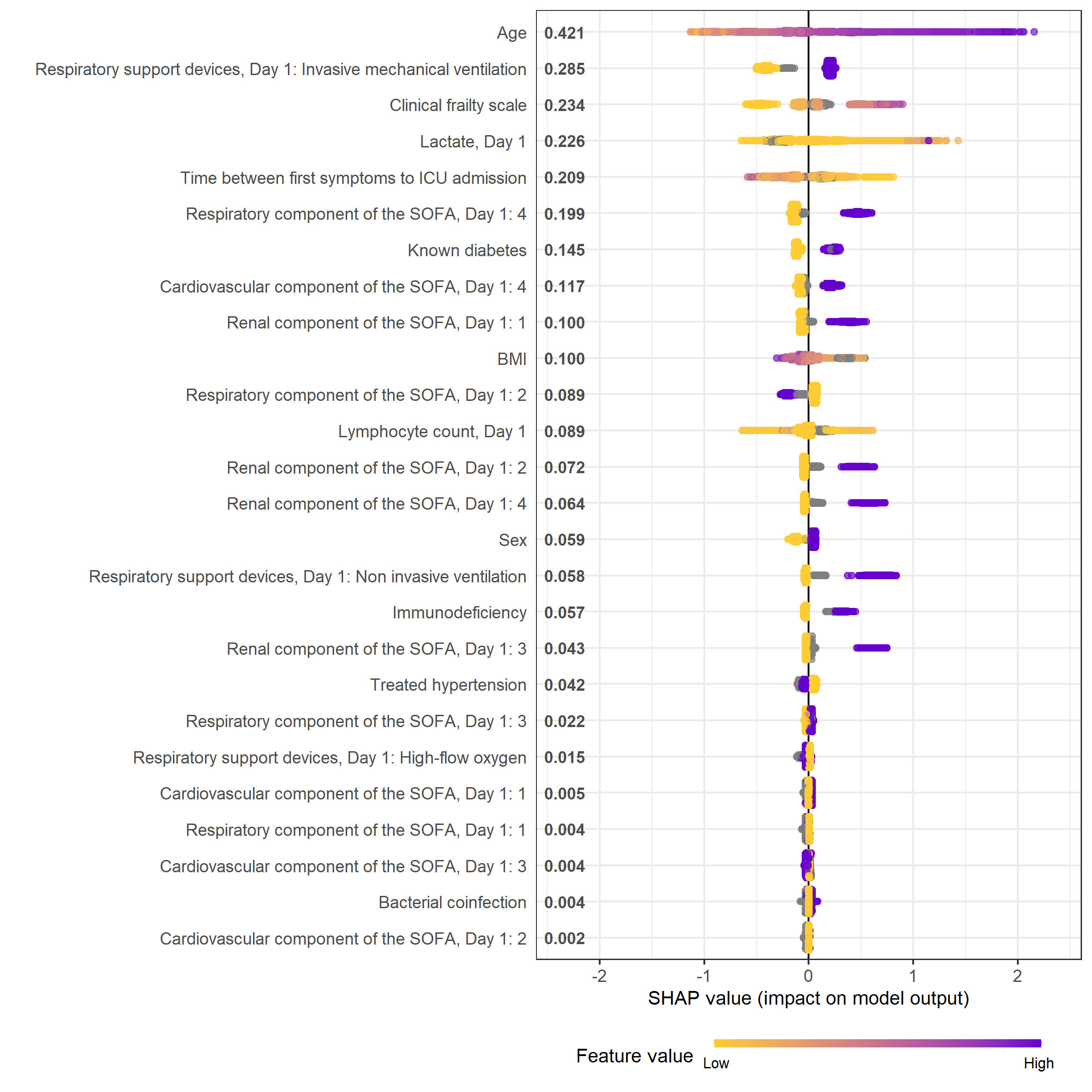
***

**SOCIC-7 :**

**
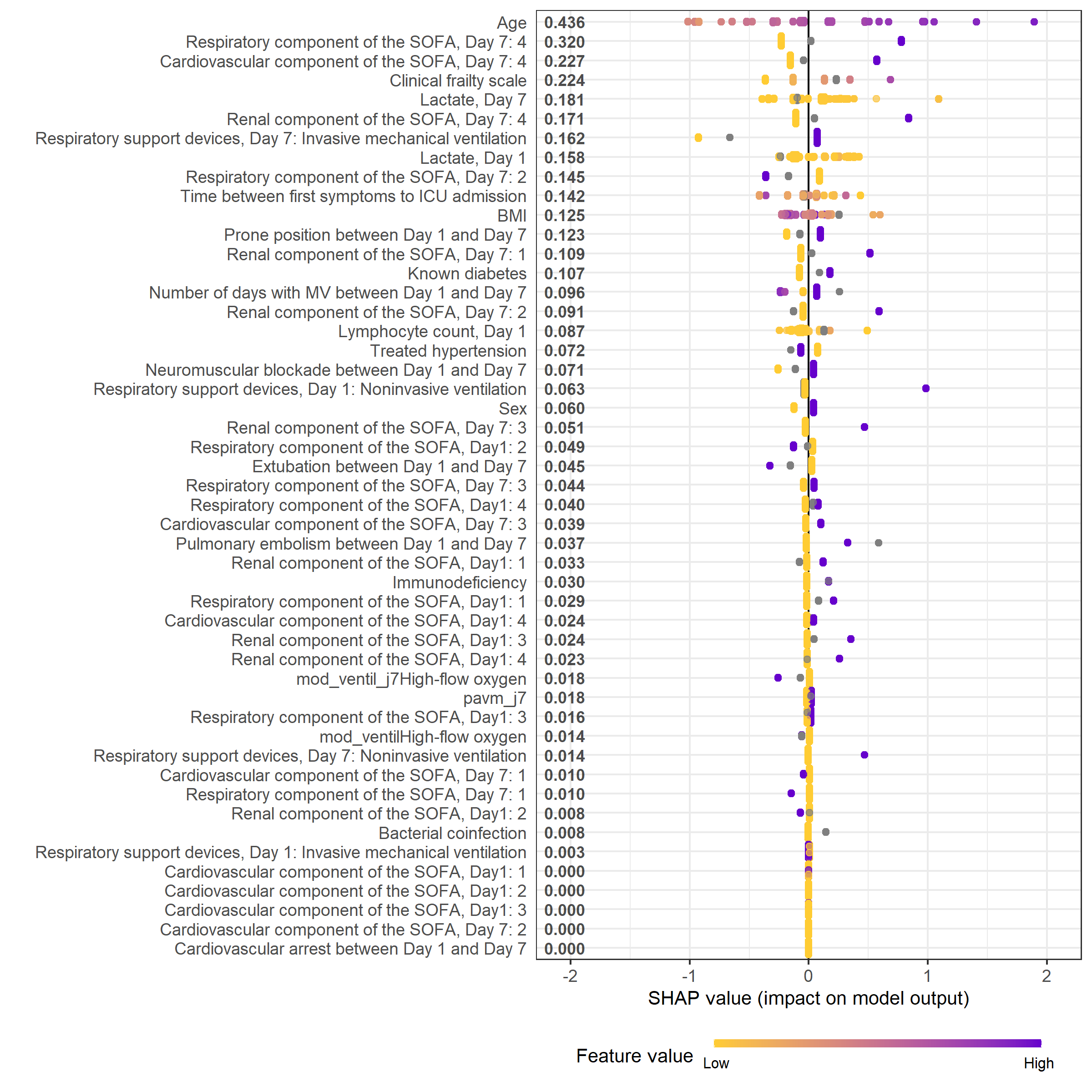
**

***SOSIC-14:***

***
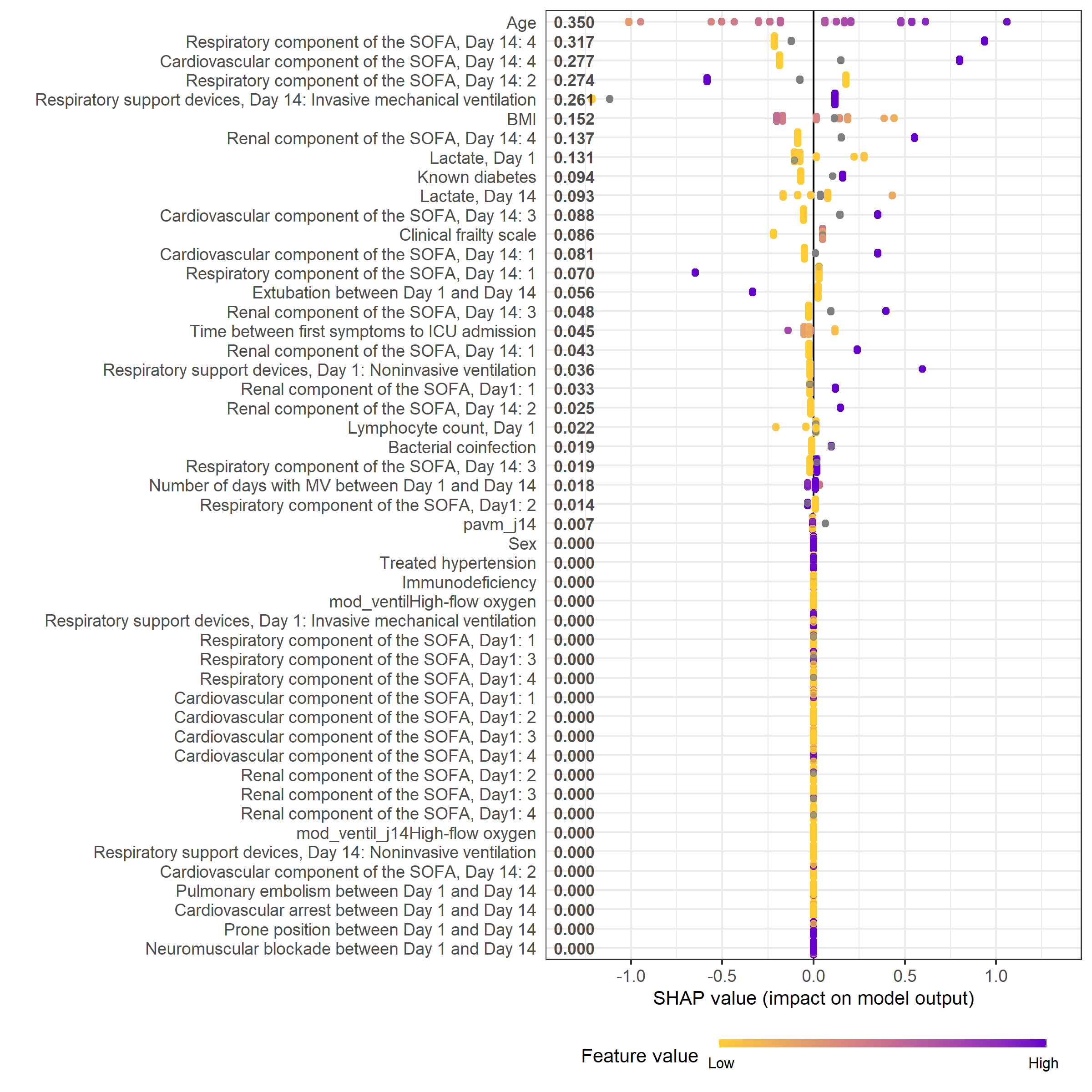
***
