# Supplementary material for: Predicting 90-day survival of patients with COVID-19: Survival of Severely Ill COVID (SOSIC) scores
Source: Ann Intensive Care. 2021 Dec 11;11:170. doi: 10.1186/s13613-021-00956-9 (PMC8665857; doi:10.1186/s13613-021-00956-9)
Supplement: Supplementary file 3 — Additional file 3. Validation Metrics of the SOSIC-1, SOSIC-7, and SOSIC-14 Scores. [file 13613_2021_956_MOESM3_ESM.docx]

**Additional file 3. Validation Metrics of the SOSIC-1, SOSIC-7, and SOSIC-14 Scores**

| **Validation** | **SOSIC-1** | **SOSIC-7** | **SOSIC-14** |
| --- | --- | --- | --- |
| **Apparent** |  |  |  |
| AUC | 0.82 (0.80-0.83) | 0.83 (0.81-0.85) | 0.85 (0.83-0.87) |
| Brier score | 0.153 (0.147-0.159) | 0.146 (0.140-0.154) | 0.133 (0.125-142) |
| **Bootstrap^a^** |  |  |  |
| AUC | 0.74 | 0.77 | 0.79 |
| Brier score | 0.237 | 0.221 | 0.198 |
| **Test dataset** |  |  |  |
| AUC | 0.76 (0.71-0.81) | 0.80 (0.74-0.86) | 0.76 (0.68-0.83) |
| Brier score | 0.173 (0.153-0.193) | 0.147 (0.125-0.172) | 0.158 (0.129-0.191) |

*SOSIC = Survival Of Severely Ill COVID; AUC = area under the receiver-operating characteristics curve.*

^a^95% confidence intervals of bootstrap-validated optimism-corrected performance metrics were not estimated because that would require too long computation time.
