# Supplementary material for: Predicting 90-day survival of patients with COVID-19: Survival of Severely Ill COVID (SOSIC) scores
Source: Ann Intensive Care. 2021 Dec 11;11:170. doi: 10.1186/s13613-021-00956-9 (PMC8665857; doi:10.1186/s13613-021-00956-9)
Supplement: Supplementary file 4 — Additional file 4. Correlation between SOSIC-1, SOSIC-7, and SOSIC-14. [file 13613_2021_956_MOESM4_ESM.docx]

**Additional file 4: Correlation between SOSIC-1, SOSIC-7, and SOSIC-14 in the A) development and B) the test datasets**
