# Supplementary material for: Predicting 90-day survival of patients with COVID-19: Survival of Severely Ill COVID (SOSIC) scores
Source: Ann Intensive Care. 2021 Dec 11;11:170. doi: 10.1186/s13613-021-00956-9 (PMC8665857; doi:10.1186/s13613-021-00956-9)
Supplement: Supplementary file 5 — Additional file 5. Calibration and discrimination of the Survival of Severely Ill COVID (SOSIC)-1, SOSIC-7, and SOSIC-14 scores in centers from Paris-greater area and Grand Est vs other regions, respectively. [file 13613_2021_956_MOESM5_ESM.docx]

**Additional file 5: Calibration and discrimination of the Survival Of Severely Ill COVID (SOSIC)-1, SOSIC-7, and SOSIC-14 scores in centers from Paris and greater area vs other regions, respectively.**
