# Supplementary material for: Predicting 90-day survival of patients with COVID-19: Survival of Severely Ill COVID (SOSIC) scores
Source: Ann Intensive Care. 2021 Dec 11;11:170. doi: 10.1186/s13613-021-00956-9 (PMC8665857; doi:10.1186/s13613-021-00956-9)
Supplement: Supplementary file 6 — Additional file 6. Graphic representation of the performances of A) SOSIC-7 and the SOFA at day-7, and B) SOSIC-14 and the SOFA at day-14 in the development and the test datasets. [file 13613_2021_956_MOESM6_ESM.docx]

**Additional file 7: Graphic representation of the performances of A) SOSIC-7 and the SOFA at day 7, and B) SOSIC-14 and the SOFA at day 14 in the development and the test datasets**

**A)**

**B)**
